# Neutrophil diversity and plasticity: Implications for organ transplantation

**DOI:** 10.1038/s41423-023-01058-1

**Published:** 2023-06-29

**Authors:** Junwen Qu, Jingsi Jin, Ming Zhang, Lai Guan Ng

**Affiliations:** 1 Shanghai Immune Therapy Institute, Renji Hospital, Shanghai Jiao Tong University School of Medicine, Shanghai, 200127 China; 2 Department of Urology, Renji Hospital, Shanghai Jiao Tong University School of Medicine, Shanghai, 200127 China

**Keywords:** Neutrophil, Heterogeneity, Transplantation, Neutrophils, Allotransplantation

## Abstract

Neutrophils, as the first defenders against external microbes and stimuli, are highly active and finely regulated innate immune cells. Emerging evidence has challenged the conventional dogma that neutrophils are a homogeneous population with a short lifespan that promotes tissue damage. Recent findings on neutrophil diversity and plasticity in homeostatic and disease states have centered on neutrophils in the circulation. In contrast, a comprehensive understanding of tissue-specialized neutrophils in health and disease is still lacking. This article will first discuss how multiomics advances have contributed to our understanding of neutrophil heterogeneity and diversification in resting and pathological settings. This discussion will be followed by a focus on the heterogeneity and role of neutrophils in solid organ transplantation and how neutrophils may contribute to transplant-related complications. The goal of this article is to provide an overview of the research on the involvement of neutrophils in transplantation, with the aim that this may draw attention to an underappreciated area of neutrophil research.

## Introduction

Neutrophils, the most abundant leukocytes in the circulation (50–70% in humans and 10–25% in mice [1]), play a vital role in maintaining homeostasis as the first immune responder to exogenous pathogens and stimuli. It is estimated that 1-2 × 10^11^ neutrophils are produced daily in humans and 10^7^ in mice by hematopoiesis in the bone marrow [2]. Neutrophil homeostasis is maintained through a fine-tuned balance of granulocyte production, bone marrow storage and release, and migration into the vascular compartments and remote tissues. Neutrophils exhibit a short lifespan, and their half-life is less than one day, with an estimated half-life of 19 h for human blood neutrophils [3, 4]. However, in vivo labeling has revealed an average lifespan of 5.4 days for human circulating neutrophils, which is much longer than generally accepted [5]. Thus, these results demonstrate that the exact neutrophil lifespan remains elusive, raising the possibility that neutrophil lifespan is very context dependent.

Bone marrow is the primary site for leukocyte production. By 10 to 11 weeks post-conception, neutrophils first appear in human clavicular bone marrow [6]. Neutrophil precursors are detectable in the peripheral blood by the end of the first trimester, whereas mature cells appear by 14 to 16 weeks of gestation [7]. It is widely acknowledged that neutrophil development begins with the common myeloid progenitor (CMP), which gives rise to the granulocyte-monocyte progenitor (GMP) [8]. Neutrophil development comprises two primary stages: the proliferative stage, in which GMPs differentiate into myeloblasts, promyelocytes, and myelocytes; and the nonproliferative stage, in which the proliferative precursors give rise to nonproliferative metamyelocytes, band cells, and mature neutrophils [9]. Granules are generated throughout the various stages of neutrophil development. Notably, azurophil granules indicate the transition from myeloblasts to promyelocytes; specific granules occur in the myelocyte/metamyelocyte stage; gelatinase granules are present in band cells; and secretory vesicles are detected in segmented cells [10].

**Fig. 1:**
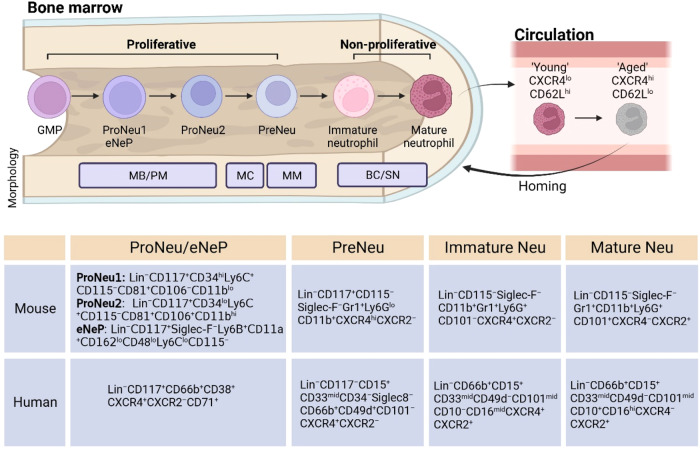
Development of neutrophils in the bone marrow. Neutrophil development begins with the granulocyte-monocyte progenitor (GMP) pool. The earliest unipotent neutrophil progenitors are termed early neutrophil progenitors (eNePs) in humans and early committed neutrophil progenitor (proNeu1) in mice. proNeu1 gives rise to proNeu2 progenitor, subsequently differentiating into a proliferative neutrophil precursor (preNeu), which finally develops into immature neutrophils and mature neutrophils. In homeostasis, CXCR4, a retention signal in the bone marrow, gradually downregulated as neutrophils mature, whereas CXCR2 delivers an egress signal and increases in abundance as neutrophils mature. Therefore, CXCR4^lo^CXCR2^hi^ neutrophils are released into the bloodstream. In the circulation, neutrophils undergo circadian ageing. Aged neutrophils (CXCR4^hi^CD62L^lo^) are demonstrated to instruct additional heterogeneity and have a role in regulating the hematopoietic stem cell niche through their clearance in the bone marrow. Furthermore, aged neutrophils are recruited back to the bone marrow with circadian frequency. MB myeloblast, PM promyelocyte, MC myelocyte, MM metamyelocyte, BC banded cell; SN segmented neutrophil

In the current paradigm, characterization of the different stages of neutrophil development is examined by Giemsa staining-based histologic examination, which reflects cell morphological features [11]. Recently, advances in multiomics techniques at single-cell resolution have greatly expanded our understanding of neutrophil ontogeny. By integrating multiple single-cell-based analyses, an early committed neutrophil progenitor (proNeu1) marked by Lin^–^CD117^+^CD34^hi^Ly6C^+^CD115^–^CD81^+^CD106^–^CD11b^lo^ that exists within GMPs gives rise to the CD117^+^CD34^lo^Ly6C^+^CD115^–^CD81^+^CD106^+^CD11b^hi^ proNeu2 progenitor subset [12], subsequently differentiating into a proliferative neutrophil precursor (preNeu) marked by Lin^–^Siglec-F^–^CD117^+^CXCR4^+^Gr1^+^CD11b^+^, which finally develops into immature neutrophils and mature neutrophils [13]. proNeu1 and preNeu rapidly expand under inflammatory stress, indicating that these populations can serve as a source for rapid neutrophil repopulation during emergency granulopoiesis. Furthermore, distinctive roles of preNeu and immature neutrophils have been revealed in murine sepsis and tumor models [13]. Notably, similar subsets of proNeu1, proNeu2 and preNeu can be detected in humans. Moreover, early unipotent human bone marrow neutrophil progenitors (eNePs), defined by the distinct surface protein markers CD71 and CD117, and murine eNePs, marked by Lin^−^CD117^+^SiglecF^−^Ly6B^+^CD11a^+^CD162^lo^CD48^lo^Ly6C^lo^CD115^−^, have been discovered and can be detected in the circulation of cancer patients and tumor-bearing mice, respectively [14, 15] (Fig. 1). Additionally, a recent study further identified a population of CD66b^−^CD64^dim^CD115^−^ cells in human bone marrow as neutrophil-committed progenitors [16]. In this study, the authors used RNA-sequencing to profile neutrophil-committed progenitors, which include promyelocytes (PMs), myelocytes (MCs), metamyelocytes (MMs), band cells (BCs), segmented neutrophils (SNs), and mature neutrophils. Importantly, the different stages of neutrophils characterized by cell morphological features indeed exhibit distinct transcriptomic signatures, which means that cell morphology and transcriptomics analyses can be integrated to precisely identify neutrophil stages. In mice, CD101 expression increases during the maturation process and serves as a robust marker for distinguishing mature neutrophils from immature neutrophils [13]. Taken together, these studies delineate the transcriptomic and protein features in neutrophil development and define the neutrophil developmental trajectory in steady-state and emergency granulopoiesis.

Mature neutrophils remain in the bone marrow for an additional 6–7 days in humans and approximately 3 days in mice [17–19], forming a reservoir called the ‘bone marrow reserve’, from which neutrophils can be rapidly mobilized in response to infection or other external stimuli [20]. Mature neutrophils are released into the bloodstream in a chemokine-regulated manner. CXCR4 provides a retention signal in the bone marrow. It is gradually downregulated as neutrophils mature, whereas CXCR2 delivers an egress signal and increases in abundance as neutrophils mature, resulting in neutrophils exiting the bone marrow [21]. There is a postmitotic pool of 5.59 ± 0.9 × 10^9^ neutrophils per kilogram of body weight in humans, from which an estimated 0.87 × 10^9^ cells per kilogram are released from the bone marrow each day [22], indicative of rapid turnover. After daily cycling, neutrophils migrate back into the bone marrow, where they are eliminated by macrophages to maintain homeostasis [21].

## Neutrophil heterogeneity

**Fig. 2:**
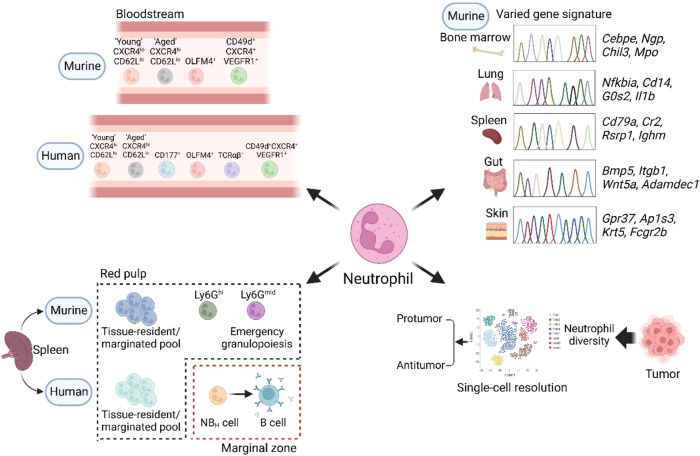
Heterogeneity of neutrophils in tissues. Emerging evidence has revealed that different neutrophil subpopulations, defined by the indicated markers, are present in the circulation under homeostatic conditions in humans and mice. However, whether these cells are real distinct functional populations remains to be further validated. Under resting conditions, neutrophils are also marginated in pools within spleen and lung, but the phenotypes and functions of these cells remain poorly defined. A unique population of human neutrophils was found in the marginal zone (MZ) of the spleen, and these were termed B cell helper neutrophils (NB_H_ cells) owing to their robust B cell-activating properties. In the murine spleen, two neutrophil subpopulations localized in the red pulp participate in emergency granulopoiesis (immotile Ly6G^mid^ immature neutrophils) and pneumococcal clearance (active-moving Ly6G^hi^ mature neutrophils). Importantly, neutrophils exhibit a marked gene signature diversity across multiple tissues such as bone marrow, lung, spleen, gut, and skin in mice, which supports the concept that specialized neutrophils exist within different homeostatic tissues. Neutrophils show altered heterogeneity in non-homeostatic states such as cancer. Recent studies have unraveled that neutrophils in tumor tissue and adjacent normal tissue are distinct and highly heterogeneous at the level of single-cell transcriptomics, with both pro- and anti-tumor functions

Emerging evidence has demonstrated the heterogeneity of neutrophils, challenging the long-held belief that neutrophils are a relatively homogeneous population. Under homeostatic conditions, most circulating neutrophils are mature neutrophils; however, neutrophil diversity may result from the equilibrium between aged circulating neutrophils and newly released neutrophils from the bone marrow. Aged neutrophils in the circulation are defined by high CXCR4 and low CD62L (also known as L-selectin) expression, and they have been demonstrated to have a role in regulating the hematopoietic stem cell niche through their clearance in the bone marrow [23, 24]. In the bone marrow, local macrophages ingest CXCR4^hi^ neutrophils and downregulate CXCL12 expression, thereby releasing CXCR4^lo^CD62L^hi^ neutrophils with a peak every 24 hours [24]. Moreover, circadian changes in the transcriptional and migratory properties in a CXCR2-dependent manner modulate the external topology of neutrophils to facilitate homeostatic egress from blood vessels at night, thus enhancing antimicrobial activity in tissues [25]. Aged neutrophils exhibit augmented integrin activation and increased neutrophil extracellular trap (NET) formation under inflammation, indicating an active phenotype. Interestingly, neutrophil aging can be induced by the gut microbiota via Toll-like receptors and MyD88-mediated signaling [26]. Recent findings have demonstrated that the development, aging, and elimination of neutrophils are accelerated in mice with a predisposition to interleukin-4 (IL-4)-mediated type 2 immunity, which, in turn, causes susceptibility to infection by several bacteria [27]. Thus, neutrophil aging modulates the compartmentalization of neutrophils diurnally to optimize their defense function while limiting collateral tissue damage under steady state. Human aged neutrophils exhibit elevated CXCR4 expression in vitro [28], despite the lack of investigations on the human neutrophil aging process. There are several subsets of neutrophils in the human peripheral circulation with unclear functions. In healthy individuals, 45–65% of circulating neutrophils express CD177, and 20–25% express the glycoprotein olfactomedin 4 (OLFM4) [29–31], with the former being increased in abundance in a variety of inflammatory diseases [32–34], while the latter is associated with sepsis [35]. In addition, TCRαβ^+^ or proangiogenic CD49d^+^CXCR4^+^VEGFR1^+^ circulating neutrophils are present in healthy persons [36, 37] (Fig. 2). However, whether these cells represent distinct functional populations or solely a transient functional state remains to be determined.

A growing number of studies have investigated the presence of neutrophil populations in different tissues. These tissue neutrophil populations can be resident or newly infiltrated, and they can change their phenotypes depending on the tissue microenvironment. Even at steady state, neutrophils can be detected at low levels in most tissues, excluding the brain and sex gonads. In contrast, the primary neutrophil pool is found in the bone marrow, spleen, and lung [2, 38]. Importantly, neutrophils exhibit marked phenotypic diversity across multiple tissues, such as bone marrow, spleen, lung, liver, thymus, kidney, brain and mesenteric lymph nodes, in mice, whereas some myeloid cells, such as plasmacytoid dendritic cells, show highly consistent phenotypes among these tissues [39], which supports the concept that specialized neutrophils exist within different homeostatic tissues. Correspondingly, the distribution of neutrophils varies in different tissues. For instance, neutrophils in the lung are in close contact with endothelial cells and circulate through tightly intertwined pulmonary capillaries [40–42]. Likewise, neutrophils in the spleen and bone marrow are mainly located in the red pulp or perivascular space, respectively, whereas intestinal neutrophils are located around isolated lymphoid follicles, and a small number of neutrophils are found in the dermis [38, 41, 43, 44]. These varied localizations within the different tissues may partly explain the neutrophil specification in those tissues. A distinct human neutrophil population found in the splenic marginal zone (MZ) is defined as ‘B-cell helper neutrophils (NB_H_ cells)’ due to their unique ability to promote B-cell proliferation and antibody production via secretion of soluble pentraxin 3 and indicated cytokines [45, 46], which is not observed in circulating neutrophils. In addition, two subsets of neutrophils, namely, active-moving Ly6G^hi^ mature neutrophils and immotile Ly6G^mid^ immature neutrophils, have been reported in the splenic red pulp, with specialized functions in regulating pneumococcal clearance and emergency granulopoiesis [44] (Fig. 2). It has been reported that neutrophils live just as long or even longer in tissues than in the circulation by using a neutrophil-specific fate mapping mouse model to track synchronous waves of neutrophils released from the bone marrow [41], thereby providing evidence to support the notion that neutrophils have sufficient time to receive and integrate cues within the tissue environment for their diversification. Importantly, a recent study has provided a description of human neutrophil diversity in normal and stress granulopoiesis [47]. However, the heterogeneity of neutrophils in different tissues has not been thoroughly elucidated, nor has the mode of regulation of tissue neutrophil diversity. Exploring these issues is of great significance for understanding neutrophil biology.

Neutrophils show altered heterogeneity in nonhomeostatic states, with rapidly changing phenotypic and functional properties. With the development of scRNA sequencing technology, neutrophils can now be efficiently captured for analysis, a previously challenging task. Recent studies have revealed that neutrophils in tumor tissue and adjacent normal tissue are distinct and highly heterogeneous at the level of single-cell transcriptomics. The transcriptomic signatures of tumor-associated neutrophils (TANs) and adjacent normal tissue-associated neutrophils (NANs) have been dissected in non-small cell lung carcinoma, and TANs can be further divided into four subpopulations that may acquire specific functional properties. Moreover, a tissue-resident neutrophil-derived gene signature is associated with immune checkpoint blockade failure [48]. Neutrophil clusters with distinct gene signatures were identified in a separate study, supporting the notion that transcriptional changes may coordinate neutrophil differentiation in space and time [49] (Fig. 2). We envision that the workflow established for examining neutrophils in tumor tissues will be a solid foundation for future research into neutrophil function in transplantation.

In addition, neutrophils exhibit diversity in aging, autoimmune diseases such as systemic lupus erythematosus, cardiovascular diseases such as atherosclerosis and myocardial infarction, nervous system diseases such as degenerative diseases and stroke, and respiratory diseases such as COVID-19, chronic obstructive pulmonary disease, lung fibrosis, tuberculosis, or asthma [2, 31, 50–54]. Notably, most studies have identified neutrophil subpopulations in a simple manner by utilizing typical neutrophil lineage, maturation, or activation markers. A deeper understanding of the intrinsic properties of these neutrophil subpopulations, including their transcriptomic and phenotypic characteristics in the tissue context, is necessary for elucidating the origin of neutrophil diversification.

## Neutrophil functional plasticity: tissue injury and tissue repair

Neutrophils are involved in promoting the resolution of inflammation and facilitating tissue repair in addition to their well-characterized proinflammatory function. There are three strategies adopted by neutrophils to promote tissue repair [55, 56]: 1) engulfing damaged cells and removing cellular debris; 2) after activation, neutrophils can rapidly release their intracellular storage of growth factors and pro-angiogenic factors to stimulate tissue regeneration and angiogenesis; and 3) apoptotic neutrophils release ‘find me’ and ‘eat me’ signals to attract macrophages for efferocytosis. Revascularization is a key part of the repair process following tissue injury, and neutrophils have been found to promote vascular remodeling in isolated pancreatic islet transplantation and late-stage sterile inflammation [57, 58]. Neutrophils are able to decontaminate the wound from foreign debris and defend against potential pathogens, thereby promoting efficient wound repair [59]. Furthermore, neutrophils also contribute to muscle growth and repair, nerve regeneration following ocular injury, healing responses after myocardial infarction, and lung epithelial cell proliferation in a model of acute lung injury and can accelerate inflammatory resolution in acute intestinal infection [55, 60–62]. Neutrophil proinflammatory or anti-inflammatory properties are tightly regulated processes; any disruption of this equilibrium can easily lead to a severe infection or tissue fibrosis. In the following sections, we will examine the role of neutrophils in solid organ transplantation, where complications such as ischemia-reperfusion injury, rejection, and delayed graft function may be caused by neutrophil dysfunction, as well as potential neutrophil-based strategies for enabling normal graft function.

## Neutrophils in ischemia-reperfusion injury

Substantial gaps remain between the demands for transplant organs and supply from donors. To bridge the huge gap related to organ shortage and shorten the transplant waiting time for patients with end-stage disease, the proportion of marginal organs procured from extended criteria donors (ECDs) or donation after cardiac death (DCD) has increased considerably. However, organs from these donors tend to be more susceptible to ischemia-reperfusion injury (IRI), an inevitable pathogenic event following transplantation that can subsequently lead to worse outcomes.

The accumulation of neutrophils in the kidney has been described both in animal models and in human acute kidney injury (AKI). Neutrophils are primary responders that are recruited from the circulation to the sites of injury. The proportion of neutrophils is increased, particularly in the renal interstitium, as early as 30 min after ischemia-reperfusion and reaches a peak at 24 h [63, 64]. However, the role of neutrophils in renal IRI remains elusive since some studies have failed to find a protective effect on ischemic AKI by using neutrophil blockade or depletion [65, 66]. Neutrophils are recruited from the vasculature into inflamed tissues through a series of behaviors, such as tethering, rolling, adherence, crawling and ultimately transmigration [67]. The attachment of neutrophils to inflamed tissue is dependent on interactions with adhesion molecules [68]. In renal IRI, previous studies have demonstrated the role of P-selectin, E-selectin, intercellular adhesion molecule-1 (ICAM-1), integrin and CD44 in neutrophil recruitment. Blocking the molecules related to neutrophil-endothelial adhesion has a protective effect in renal IRI [69–73]. A recent study identified DPEP1 as a neutrophil adhesion receptor that plays a major role in neutrophil recruitment in conjunction with CD44 and ICAM-1, and targeting DPEP1 may be a promising strategy for alleviating renal IRI [74]. During IRI, neutrophils adhere to the vascular endothelium in the renal medulla, causing plugging in the microvasculature [75]. Once neutrophils degranulate, proteases, myeloperoxidases (MPOs), cytokines and oxygen free radicals are released and can participate in the induction of renal injury [76].

Neutrophils can form neutrophil extracellular traps (NETs), which are web-like structures composed of DNA, histones, and antimicrobial peptides such as MPO, elastase and cathepsin G [77, 78]. NETs were initially described to play a dominant role in antimicrobial defense [79]. A recent study uncovered the function of NETs in sterile inflammation, particularly in IRI. During ischemic renal injury, neutrophils infiltrate the renal interstitium and release cytotoxic histones while undergoing NET formation, exacerbating tubular epithelial cell injury and interstitial inflammation [80]. The interaction between platelets and neutrophils causes NET formation, leading to a further increase in renal inflammation and tissue damage. In a recent study, pretreatment with clopidogrel resulted in renoprotection by limiting platelet aggregation prior to renal ischemia and subsequently reducing the formation of NETs in renal tissue in mice [81]. In addition, P2RX1, a purinergic receptor, is involved in NET formation in renal IRI tissue by inducing platelet and neutrophil metabolic interactions [77]. Peptidyl arginine deaminase (PAD)-4, which converts arginine to citrulline post-translationally, is a critical stage in NET formation and causes inflammation after renal IRI. Mice lacking PAD-4 have fewer NETs and reduced levels of proinflammatory cytokines, which helps to alleviate renal IRI [82]. Moreover, treatment with DNase I results in improved recovery of renal function and attenuated renal IRI by accelerating the clearance of intrarenal DNA debris [83]. Therefore, these findings support the notion that neutrophil NETosis is a crucial process in IRI.

**Fig. 3:**
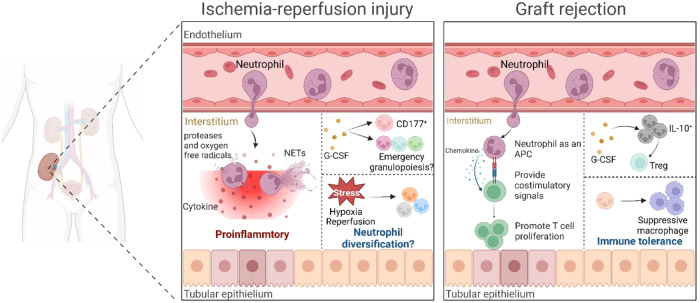
The heterogeneity of neutrophils in ischemia-reperfusion injury and graft rejection. Following renal IRI, neutrophils are recruited to the site of IRI and subsequently marginate to the vascular endothelium. Once neutrophils degranulate, cytokines, proteases and oxygen free radicals secreted can participate in the induction of renal injury. Neutrophil NETosis leads to a further increase in renal inflammation and tissue damage. Renal IRI enhances G-CSF expression in the kidney and modulates emergency granulopoiesis, also inducing CD177^+^ neutrophil population in humans. Neutrophils under the stress such as hypoxia and reperfusion, as well as under the influence of inflammatory mediators in the context of IRI may play distinct roles at various phases of the IR response, thus may result in neutrophil heterogeneity in renal IRI. In graft rejection, neutrophils can secret T cell chemokines and subsequently prime the CD8^+^ T cells. On the other hand, neutrophils serve as an APC. Neutrophils cross-prime CD4^+^ T cells via MHCII and cross-present to CD8^+^ T cells via MHCI. G-CSF can induce suppressor IL-10^+^ neutrophils to promote regulatory T cells (Treg). In addition, neutrophils also interconnect with other immune cells such as suppressive macrophages to induce immune tolerance, thereby alleviating graft rejection

Neutrophils are increasingly recognized as having the capacity to diversify [84], particularly in response to stress or disease conditions. However, it remains unclear how neutrophil heterogeneity and diversification may contribute to IRI. In human renal allografts, ischemic injury induces a transient increase in neutrophil surface glycoprotein CD177 that correlates with renal tubular epithelial G-CSF levels. I/R can induce the upregulation of G-CSF expression in a mouse model of renal IRI and can induce neutrophilia [85, 86]. In addition, a significantly higher serum concentration of G-CSF with increased neutrophils in the graft and periphery is detected following transplantation of murine lung allografts after prolonged cold ischemic storage [87]. It is well established that G-CSF stimulates neutrophil production and release from the bone marrow in both preclinical and clinical settings [88]. A recent study demonstrated that G-CSF treatment in human patients resulted in an increased number of preNeu and immature neutrophils in the peripheral blood [47]. In stress-induced granulopoiesis, committed precursors and immature neutrophils expand and are released prematurely into the blood, and they coexist with the terminally differentiated mature neutrophils. With their ability to diversify in response to the local milieu, neutrophils under the influence of inflammatory mediators in the context of IRI may play distinct roles at various phases of the IR response (Fig. 3). Thus, understanding the precise mechanism of neutrophil differentiation during IRI may be key to developing novel therapies.

## Neutrophils highly correlate with delayed graft function

Delayed graft function (DGF) is a common complication of kidney transplantation and is associated with worse clinical outcomes. DGF is defined as the need for dialysis within the first week following transplantation. Ischemia-reperfusion injury and immune responses within transplants are recognized as major causes of DGF [89]. Turunen et al. reported that neutrophil infiltration is the most important predictor of DGF [90]. Furthermore, a precise DGF and long-term graft survival predictive strategy was established based on NET-related genes, indicating the importance of neutrophil function in DGF [91]. The inflammatory environment in donor kidneys induces the upregulation of cell adhesion molecules such as ICAM-1 and P-selectin, which facilitates neutrophil recruitment that becomes a risk factor for DGF. P-Selectin expression correlates with neutrophil infiltration and DGF [92]. A phase I clinical trial in 18 recipients of cadaveric renal allografts using anti-ICAM-1 monoclonal antibody showed a lower incidence of DGF [93], whereas enlimomab, another anti-ICAM-1 antibody, failed to reduce DGF incidence, as revealed by a randomized controlled trial [94]. A recent study showed that dynamic changes in the neutrophil-to-lymphocyte ratio in donors are promising in predicting posttransplant DGF, which can assist in the early recognition and management of renal graft dysfunction [95].

## Neutrophil and graft rejection

Neutrophils play a pivotal role in the initiation of acute cellular rejection. In a murine model of cardiac allograft rejection, blocking or lacking the neutrophil chemokine receptor CXCR2 significantly reduces neutrophil infiltration and inhibits T-cell infiltration into cardiac grafts. In addition, the combination of costimulatory blockade with peritransplant neutrophil depletion or anti-CXCL1/2 antibodies greatly enhances cardiac allograft survival, indicating that early neutrophil-mediated tissue damage may promote T-cell-mediated rejection [96]. Another study demonstrated that neutrophil depletion markedly attenuates acute allograft rejection mediated by memory CD8^+^ T cells and allows alloreactive regulatory T cells (Tregs) to maintain long-term allograft survival [97]. Fas ligand and perforin expressed by infiltrating neutrophils can result in the induction of T-cell chemoattractants, including CCL1, CCL2 and CCL5, and subsequently prime CD8^+^ T cells [98]. On the other hand, it is increasingly appreciated that neutrophils are capable of cross-priming CD4^+^ T cells via major histocompatibility complex II (MHCII) and cross-presenting to CD8^+^ T cells via MHCI, suggesting that neutrophils could also serve as antigen-presenting cells (APCs) [99, 100] (Fig. 3). Specifically, after interferon (IFN)-γ stimulation, the expression of MHCII and costimulatory molecules of neutrophils is significantly upregulated, thus facilitating neutrophils to prime Th1 and Th17 differentiation [101]. In a mouse orthotopic lung transplant model, infiltrating neutrophils stimulate donor-derived dendritic cells within lung allografts immediately after reperfusion in a contact-dependent manner and promote IL-12 production by DCs, which leads to enhanced Th1 alloimmunity and graft rejection [87].

In organ transplantation, the role of neutrophils is commonly related to antibody-mediated rejection [102]. It was previously reported that antibody-mediated rejection induces neutrophil infiltration and activation that participate in the injury of allograft tissues in mouse heart and lung transplant models [103, 104]. Neutrophils can express Fcγ receptors (FcγR), such as FcγRI, FcγRIIA and FcγRIIIB. FcγR-mediated direct cellular activation is associated with donor-specific antibody-induced downstream immune activation, which is involved in antibody-mediated rejection (AMR) of kidney transplantation [105]. A previous study suggested that FcγR-mediated immune recognition of MHC Ab bound to endothelium and the subsequent neutrophil activation within grafts are responsible for the generation of anti-MHCI antibody-mediated acute lung injury in a mouse model [106]. FcγRIII-deficient cardiac allograft recipients undergo an acceleration of rejection accompanied by prominent perivascular margination of monocytes and neutrophils, increased alloantibody production, activation of C4d deposition and extensive accumulation of apoptotic cells [107]. In a mouse model of acute antibody-mediated rejection, neutrophil infiltration into kidney allografts in CCR5-deficient recipients was detected on Day 3 after transplantation and decreased as ischemic renal injury was attenuated, and a later surge of neutrophil infiltration occurred again as DSA titers increased [108]. Prior studies have elucidated that a higher level of NETs and netting neutrophils in biopsies are associated with the development of acute antibody-mediated rejection in kidney transplants [109]. In addition, neutrophils promote the survival and differentiation of B cells and plasma cells and facilitate immunoglobulin class switching and antibody production by producing B-cell-activating factor and a proliferation-inducing ligand (APRIL) [45]. In addition, it has been reported that NETs can serve as a novel, reliable, and simple-to-measure biomarker for predicting the outcomes of lung transplant recipients [110]. These findings suggest that neutrophil subsets or functional alterations are associated with patient outcomes following kidney transplantation, necessitating additional research. Future research should concentrate on determining at which hierarchical level are neutrophils involved in AMR in the kidney, i.e., by establishing a local microenvironment at the local site; or by exerting a direct effect on T and B cells at the local site; or by secreting a soluble factor that could be released into the circulation for lymphocyte priming?

## Neutrophils and tolerance

It has long been recognized that neutrophils, as key mediators of ischemia-reperfusion injury, participate in early posttransplant innate immune responses, enhancing adaptive alloimmunity and ultimately impacting transplant outcomes. However, with the increased appreciation of the heterogeneity and diversity of neutrophils, it is important to assess whether neutrophil subpopulations that may alleviate graft injury and prevent rejection or re-establishment of tissue homeostasis exist after transplantation. Neutrophil-induced inhibition of T-cell proliferation might be essential to limit T-cell activation and maintain tolerance in inflammatory conditions. Studies have reported subsets of neutrophils that can suppress T-cell activation [111, 112]. G-CSF treatment-induced suppressive G-Neutrophils can reduce acute graft-versus-host disease in an IL-10- and Treg-dependent manner [113]. However, whether such a therapeutic strategy shows a protective effect on graft survival in solid organ transplantation needs further exploration. Neutrophils also play a key role in immunological tolerance in graft tissues by interconnecting with other immune cells. Neutrophils have been found to induce the polarization of Ly6C^lo^ suppressive macrophages by producing CSF1, thereby promoting transplant tolerance in the context of CD40L blockade [114] (Fig. 3). Neutrophil apoptosis is involved in inducing immune tolerance. Apoptotic neutrophils can be recognized by macrophages for efferocytosis by expressing ‘find me’ or ‘eat me’ signals, which have been reported to exhibit immunomodulatory functions [55]. Targeting these signals may lead to the development of therapeutic approaches for graft protection.

## Concluding remarks

Neutrophils, a fundamental cellular component of innate immunity, are crucial in a wide range of protective and immune regulatory responses. Advances in multiomics technologies have helped in recognizing and understanding neutrophil heterogeneity, thus challenging the conventional view that neutrophils are a homogeneous population. Although some specialized neutrophil subpopulations have been described in homeostatic and pathological conditions, there is still a lack of precise criteria to define these neutrophil subsets at multilayered levels, including genomic, epigenomic, and protein features and functional phenotypes. In addition, existing studies have mainly explored the heterogeneity of neutrophils from the perspective of the single-cell transcriptome, which ignores the complex spatial information of neutrophil subpopulations and is unable to elucidate the interaction between neutrophil subsets and other cells in the context of the native tissue microenvironment.

The adoption of tissue-specific phenotypes by neutrophils suggests that tissue niche cues may differentially condition neutrophils. Using multidimensional analytic techniques, it is evident that neutrophils also possess tissue-specific properties, similar to macrophages, which have been extensively studied for their tissue-specific characteristics in the past. Notably, tissue neutrophil heterogeneity and diversification studies are primarily conducted using preclinical mouse models; therefore, more investigations are required to better understand the diversity and plasticity of neutrophils in human tissues under healthy and pathological conditions, which is vital for comprehending the regulation of immune homeostasis. Therefore, we can reconsider the role of neutrophils from the point of view of single ‘microbial killers’ to include highly complex and finely regulated populations with diverse functions. We propose that neutrophil function could be ‘reprogrammed’ in numerous diseases, such as organ transplant complications. Exploration of the dynamic changes in the heterogeneity and plasticity of neutrophils under steady conditions and during disease development may lead to discovering novel therapeutic strategies that can ‘redirect’ neutrophil function back to a healthy state.
